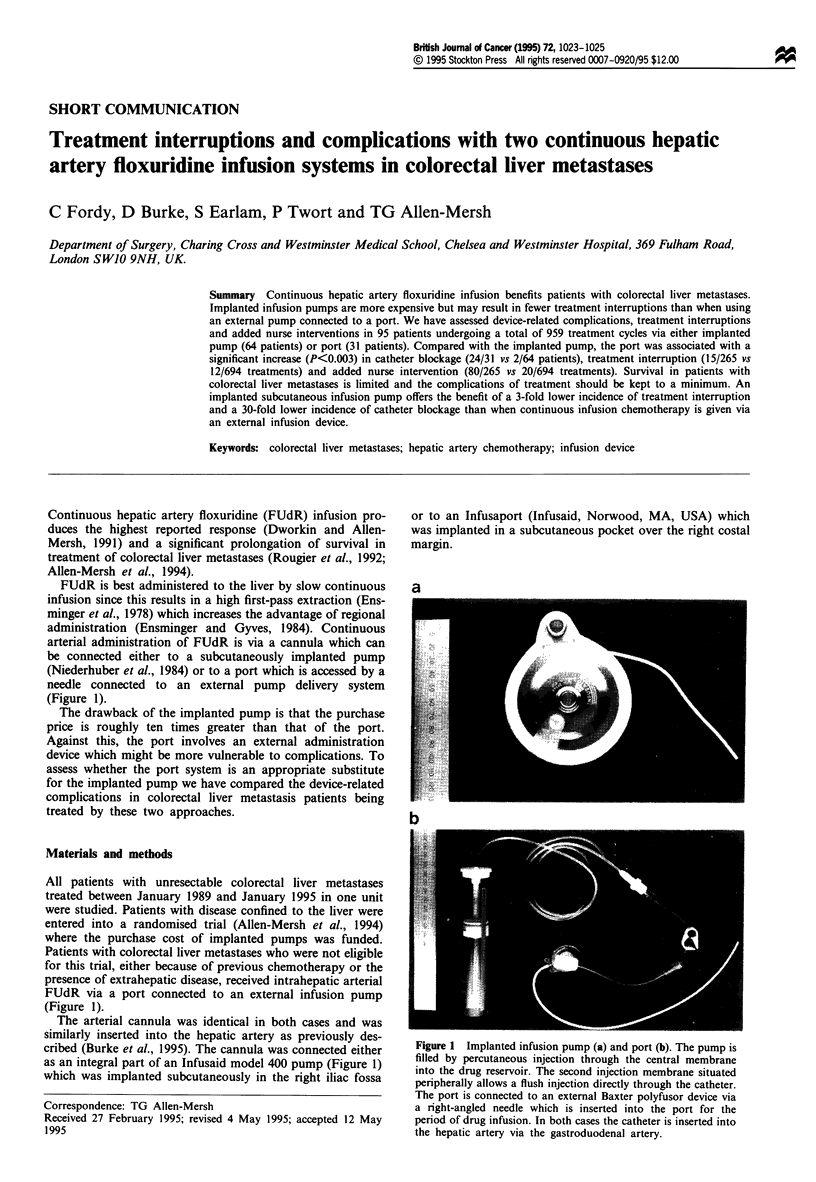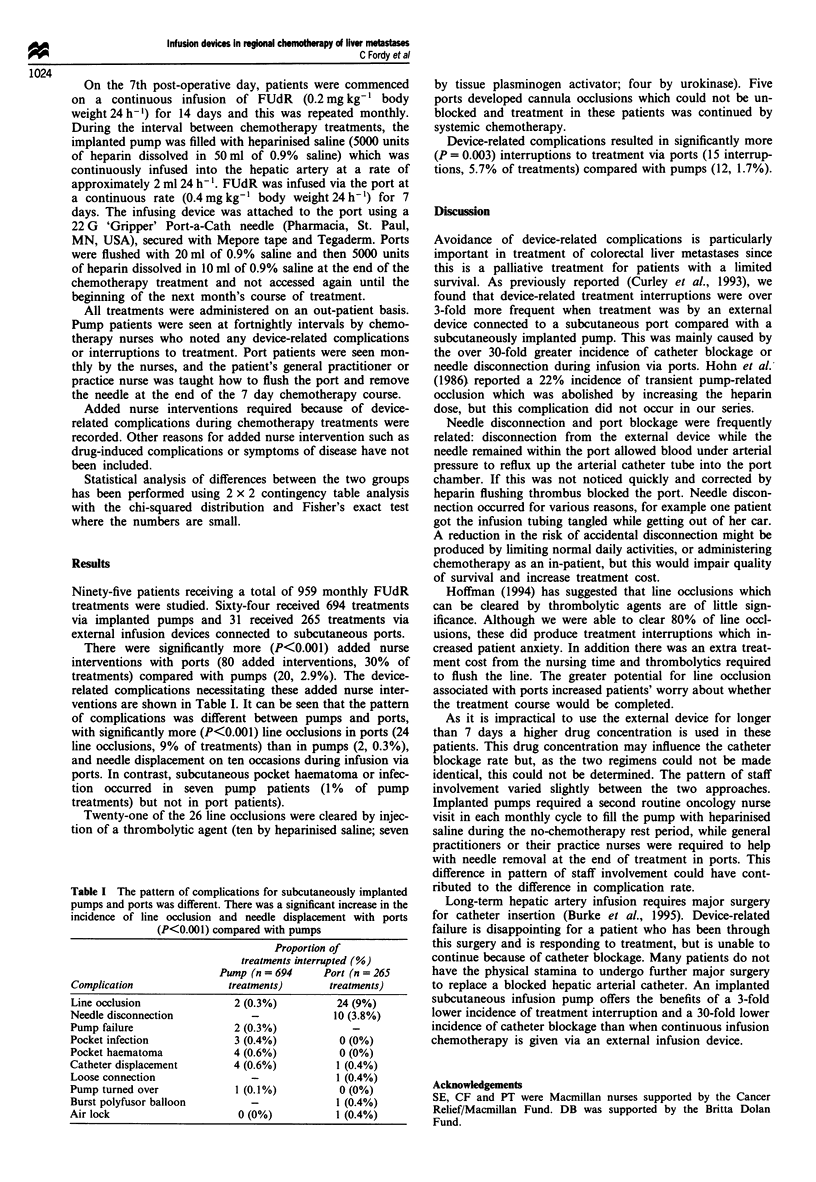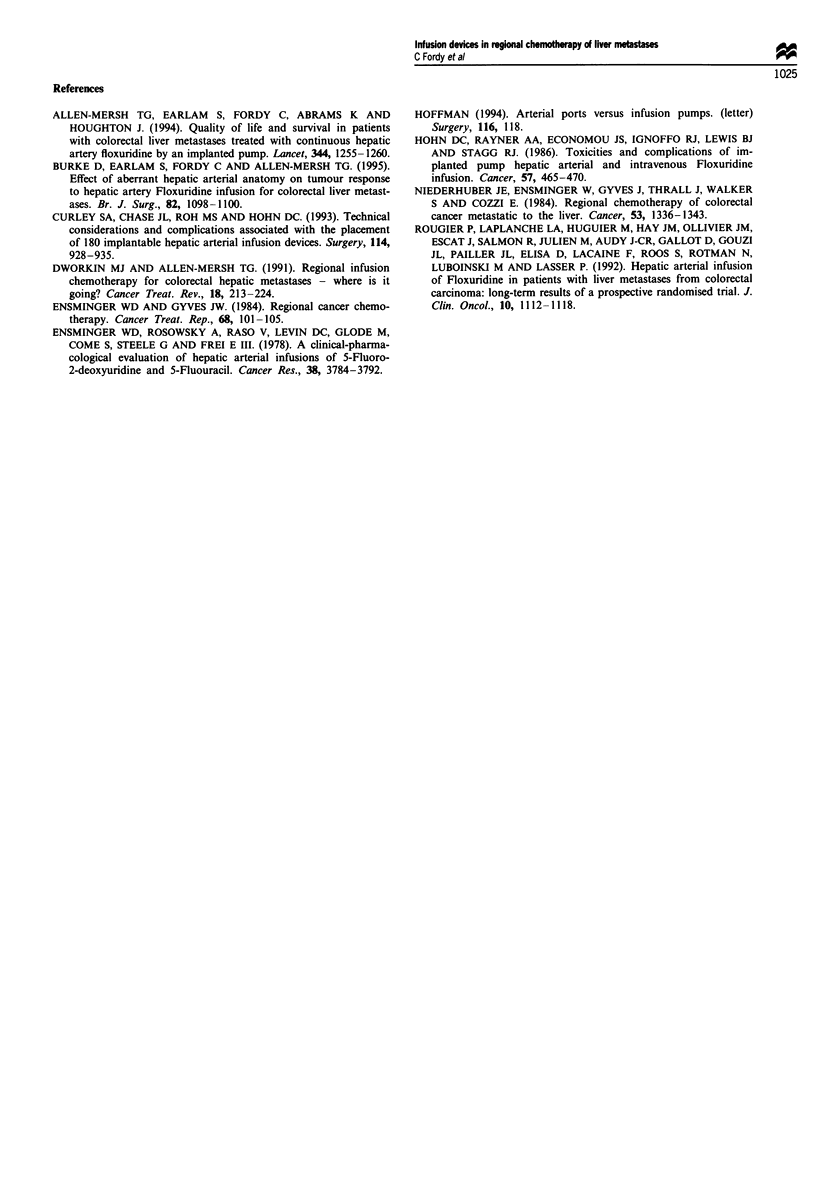# Treatment interruptions and complications with two continuous hepatic artery floxuridine infusion systems in colorectal liver metastases.

**DOI:** 10.1038/bjc.1995.455

**Published:** 1995-10

**Authors:** C. Fordy, D. Burke, S. Earlam, P. Twort, T. G. Allen-Mersh

**Affiliations:** Department of Surgery, Charing Cross and Westminster Medical School, Chelsea and Westminster Hospital, London, UK.

## Abstract

**Images:**


British Journal of Cancer (1995) 72, 1023–1025
© 1995 Stockton Press All rights reserved 0007–0920/95 $12.00

SHORT COMMUNICATION

### Treatment interruptions and complications with two continuous hepatic artery floxuridine infusion systems in colorectal liver metastases

C Fordy, D Burke, S Earlam, P Twort and TG Allen-Mersh

*Department of Surgery, Charing Cross and Westminster Medical School, Chelsea and Westminster Hospital, 369 Fulham Road, London SW10 9NH, UK.*

**Summary** Continuous hepatic artery floxuridine infusion benefits patients with colorectal liver metastases. Implanted infusion pumps are more expensive but may result in fewer treatment interruptions than when using an external pump connected to a port. We have assessed device-related complications, treatment interruptions and added nurse interventions in 95 patients undergoing a total of 959 treatment cycles via either implanted pump (64 patients) or port (31 patients). Compared with the implanted pump, the port was associated with a significant increase ($P<0.003$) in catheter blockage (24/31 *vs* 2/64 patients), treatment interruption (15/265 *vs* 12/694 treatments) and added nurse intervention (80/265 *vs* 20/694 treatments). Survival in patients with colorectal liver metastases is limited and the complications of treatment should be kept to a minimum. An implanted subcutaneous infusion pump offers the benefit of a 3-fold lower incidence of treatment interruption and a 30-fold lower incidence of catheter blockage than when continuous infusion chemotherapy is given via an external infusion device.

**Keywords:** colorectal liver metastases; hepatic artery chemotherapy; infusion device

Continuous hepatic artery floxuridine (FUdR) infusion produces the highest reported response (Dworkin and Allen-Mersh, 1991) and a significant prolongation of survival in treatment of colorectal liver metastases (Rougier *et al.*, 1992; Allen-Mersh *et al.*, 1994).

FUdR is best administered to the liver by slow continuous infusion since this results in a high first-pass extraction (Ensminger *et al.*, 1978) which increases the advantage of regional administration (Ensminger and Gyves, 1984). Continuous arterial administration of FUdR is via a cannula which can be connected either to a subcutaneously implanted pump (Niederhuber *et al.*, 1984) or to a port which is accessed by a needle connected to an external pump delivery system (Figure 1).

The drawback of the implanted pump is that the purchase price is roughly ten times greater than that of the port. Against this, the port involves an external administration device which might be more vulnerable to complications. To assess whether the port system is an appropriate substitute for the implanted pump we have compared the device-related complications in colorectal liver metastasis patients being treated by these two approaches.

### Materials and methods

All patients with unresectable colorectal liver metastases treated between January 1989 and January 1995 in one unit were studied. Patients with disease confined to the liver were entered into a randomised trial (Allen-Mersh *et al.*, 1994) where the purchase cost of implanted pumps was funded. Patients with colorectal liver metastases who were not eligible for this trial, either because of previous chemotherapy or the presence of extrahepatic disease, received intrahepatic arterial FUdR via a port connected to an external infusion pump (Figure 1).

The arterial cannula was identical in both cases and was similarly inserted into the hepatic artery as previously described (Burke *et al.*, 1995). The cannula was connected either as an integral part of an Infusaid model 400 pump (Figure 1) which was implanted subcutaneously in the right iliac fossa or to an Infusaport (Infusaid, Norwood, MA, USA) which was implanted in a subcutaneous pocket over the right costal margin.

**Figure 1** Implanted infusion pump (**a**) and port (**b**). The pump is filled by percutaneous injection through the central membrane into the drug reservoir. The second injection membrane situated peripherally allows a flush injection directly through the catheter. The port is connected to an external Baxter polyfusor device via a right-angled needle which is inserted into the port for the period of drug infusion. In both cases the catheter is inserted into the hepatic artery via the gastroduodenal artery.

Correspondence: TG Allen-Mersh
Received 27 February 1995; revised 4 May 1995; accepted 12 May 1995

On the 7th post-operative day, patients were commenced on a continuous infusion of FUdR ($0.2\,mg\,kg^{-1}$ body weight $24\,h^{-1}$) for 14 days and this was repeated monthly. During the interval between chemotherapy treatments, the implanted pump was filled with heparinised saline (5000 units of heparin dissolved in 50 ml of 0.9% saline) which was continuously infused into the hepatic artery at a rate of approximately $2\,ml\,24\,h^{-1}$. FUdR was infused via the port at a continuous rate ($0.4\,mg\,kg^{-1}$ body weight $24\,h^{-1}$) for 7 days. The infusing device was attached to the port using a 22 G 'Gripper' Port-a-Cath needle (Pharmacia, St. Paul, MN, USA), secured with Mepore tape and Tegaderm. Ports were flushed with 20 ml of 0.9% saline and then 5000 units of heparin dissolved in 10 ml of 0.9% saline at the end of the chemotherapy treatment and not accessed again until the beginning of the next month's course of treatment.

All treatments were administered on an out-patient basis. Pump patients were seen at fortnightly intervals by chemotherapy nurses who noted any device-related complications or interruptions to treatment. Port patients were seen monthly by the nurses, and the patient's general practitioner or practice nurse was taught how to flush the port and remove the needle at the end of the 7 day chemotherapy course.

Added nurse interventions required because of device-related complications during chemotherapy treatments were recorded. Other reasons for added nurse intervention such as drug-induced complications or symptoms of disease have not been included.

Statistical analysis of differences between the two groups has been performed using 2 × 2 contingency table analysis with the chi-squared distribution and Fisher's exact test where the numbers are small.

### Results

Ninety-five patients receiving a total of 959 monthly FUdR treatments were studied. Sixty-four received 694 treatments via implanted pumps and 31 received 265 treatments via external infusion devices connected to subcutaneous ports.

There were significantly more ($P<0.001$) added nurse interventions with ports (80 added interventions, 30% of treatments) compared with pumps (20, 2.9%). The device-related complications necessitating these added nurse interventions are shown in Table I. It can be seen that the pattern of complications was different between pumps and ports, with significantly more ($P<0.001$) line occlusions in ports (24 line occlusions, 9% of treatments) than in pumps (2, 0.3%), and needle displacement on ten occasions during infusion via ports. In contrast, subcutaneous pocket haematoma or infection occurred in seven pump patients (1% of pump treatments) but not in port patients).

Twenty-one of the 26 line occlusions were cleared by injection of a thrombolytic agent (ten by heparinised saline; seven by tissue plasminogen activator; four by urokinase). Five ports developed cannula occlusions which could not be unblocked and treatment in these patients was continued by systemic chemotherapy.

Device-related complications resulted in significantly more ($P = 0.003$) interruptions to treatment via ports (15 interruptions, 5.7% of treatments) compared with pumps (12, 1.7%).

**Table I** The pattern of complications for subcutaneously implanted pumps and ports was different. There was a significant increase in the incidence of line occlusion and needle displacement with ports ($P<0.001$) compared with pumps

| Complication | *Proportion of treatments interrupted (%)* Pump (n = 694 treatments) | Port (n = 265 treatments) |
|---|---|---|
| Line occlusion | 2 (0.3%) | 24 (9%) |
| Needle disconnection | – | 10 (3.8%) |
| Pump failure | 2 (0.3%) | – |
| Pocket infection | 3 (0.4%) | 0 (0%) |
| Pocket haematoma | 4 (0.6%) | 0 (0%) |
| Catheter displacement | 4 (0.6%) | 1 (0.4%) |
| Loose connection | – | 1 (0.4%) |
| Pump turned over | 1 (0.1%) | 0 (0%) |
| Burst polyfusor balloon | – | 1 (0.4%) |
| Air lock | 0 (0%) | 1 (0.4%) |

### Discussion

Avoidance of device-related complications is particularly important in treatment of colorectal liver metastases since this is a palliative treatment for patients with a limited survival. As previously reported (Curley *et al.*, 1993), we found that device-related treatment interruptions were over 3-fold more frequent when treatment was by an external device connected to a subcutaneous port compared with a subcutaneously implanted pump. This was mainly caused by the over 30-fold greater incidence of catheter blockage or needle disconnection during infusion via ports. Hohn *et al.* (1986) reported a 22% incidence of transient pump-related occlusion which was abolished by increasing the heparin dose, but this complication did not occur in our series.

Needle disconnection and port blockage were frequently related: disconnection from the external device while the needle remained within the port allowed blood under arterial pressure to reflux up the arterial catheter tube into the port chamber. If this was not noticed quickly and corrected by heparin flushing thrombus blocked the port. Needle disconnection occurred for various reasons, for example one patient got the infusion tubing tangled while getting out of her car. A reduction in the risk of accidental disconnection might be produced by limiting normal daily activities, or administering chemotherapy as an in-patient, but this would impair quality of survival and increase treatment cost.

Hoffman (1994) has suggested that line occlusions which can be cleared by thrombolytic agents are of little significance. Although we were able to clear 80% of line occlusions, these did produce treatment interruptions which increased patient anxiety. In addition there was an extra treatment cost from the nursing time and thrombolytics required to flush the line. The greater potential for line occlusion associated with ports increased patients' worry about whether the treatment course would be completed.

As it is impractical to use the external device for longer than 7 days a higher drug concentration is used in these patients. This drug concentration may influence the catheter blockage rate but, as the two regimens could not be made identical, this could not be determined. The pattern of staff involvement varied slightly between the two approaches. Implanted pumps required a second routine oncology nurse visit in each monthly cycle to fill the pump with heparinised saline during the no-chemotherapy rest period, while general practitioners or their practice nurses were required to help with needle removal at the end of treatment in ports. This difference in pattern of staff involvement could have contributed to the difference in complication rate.

Long-term hepatic artery infusion requires major surgery for catheter insertion (Burke *et al.*, 1995). Device-related failure is disappointing for a patient who has been through this surgery and is responding to treatment, but is unable to continue because of catheter blockage. Many patients do not have the physical stamina to undergo further major surgery to replace a blocked hepatic arterial catheter. An implanted subcutaneous infusion pump offers the benefits of a 3-fold lower incidence of treatment interruption and a 30-fold lower incidence of catheter blockage than when continuous infusion chemotherapy is given via an external infusion device.

**Acknowledgements**
SE, CF and PT were Macmillan nurses supported by the Cancer Relief/Macmillan Fund. DB was supported by the Britta Dolan Fund.

### References

ALLEN-MERSH TG, EARLAM S, FORDY C, ABRAMS K AND HOUGHTON J. (1994). Quality of life and survival in patients with colorectal liver metastases treated with continuous hepatic artery floxuridine by an implanted pump. *Lancet*, **344**, 1255–1260.

BURKE D, EARLAM S, FORDY C AND ALLEN-MERSH TG. (1995). Effect of aberrant hepatic arterial anatomy on tumour response to hepatic artery Floxuridine infusion for colorectal liver metastases. *Br. J. Surg.*, **82**, 1098–1100.

CURLEY SA, CHASE JL, ROH MS AND HOHN DC. (1993). Technical considerations and complications associated with the placement of 180 implantable hepatic arterial infusion devices. *Surgery*, **114**, 928–935.

DWORKIN MJ AND ALLEN-MERSH TG. (1991). Regional infusion chemotherapy for colorectal hepatic metastases – where is it going? *Cancer Treat. Rev.*, **18**, 213–224.

ENSMINGER WD AND GYVES JW. (1984). Regional cancer chemotherapy. *Cancer Treat. Rep.*, **68**, 101–105.

ENSMINGER WD, ROSOWSKY A, RASO V, LEVIN DC, GLODE M, COME S, STEELE G AND FREI E III. (1978). A clinical-pharmacological evaluation of hepatic arterial infusions of 5-Fluoro-2-deoxyuridine and 5-Fluouracil. *Cancer Res.*, **38**, 3784–3792.

HOFFMAN (1994). Arterial ports versus infusion pumps. (letter) *Surgery*, **116**, 118.

HOHN DC, RAYNER AA, ECONOMOU JS, IGNOFFO RJ, LEWIS BJ AND STAGG RJ. (1986). Toxicities and complications of implanted pump hepatic arterial and intravenous Floxuridine infusion. *Cancer*, **57**, 465–470.

NIEDERHUBER JE, ENSMINGER W, GYVES J, THRALL J, WALKER S AND COZZI E. (1984). Regional chemotherapy of colorectal cancer metastatic to the liver. *Cancer*, **53**, 1336–1343.

ROUGIER P, LAPLANCHE LA, HUGUIER M, HAY JM, OLLIVIER JM, ESCAT J, SALMON R, JULIEN M, AUDY J-CR, GALLOT D, GOUZI JL, PAILLER JL, ELISA D, LACAINE F, ROOS S, ROTMAN N, LUBOINSKI M AND LASSER P. (1992). Hepatic arterial infusion of Floxuridine in patients with liver metastases from colorectal carcinoma: long-term results of a prospective randomised trial. *J. Clin. Oncol.*, **10**, 1112–1118.